# Impact of the springtail's cuticle nanotopography on bioadhesion and biofilm formation *in vitro* and in the oral cavity

**DOI:** 10.1098/rsos.171742

**Published:** 2018-07-04

**Authors:** Christian Hannig, Ralf Helbig, Julia Hilsenbeck, Carsten Werner, Matthias Hannig

**Affiliations:** 1 Clinic of Operative and Pediatric Dentistry, Medical Faculty Carl Gustav Carus, Technische Universität Dresden, Fetscherstraße 74, 01307 Dresden, Germany; 2 Max Bergmann Center of Biomaterials, Leibniz-Institut für Polymerforschung Dresden e.V., Hohe Strasse 6, 01069 Dresden, Germany; 3 Clinic of Operative Dentistry, Periodontology and Preventive Dentistry, University Hospital, Saarland University, Building 73, 66421 Homburg/Saar, Germany

**Keywords:** pellicle, bioadhesion, saliva, springtail, hexapods, collembolan

## Abstract

Springtails (Collembola) have a nanostructured cuticle. To evaluate and to understand anti-biofouling properties of springtail cuticles’ morphology under different conditions, springtails, shed cuticles and cuticle replicates were studied after incubation with protein solutions and bacterial cultures using common *in vitro* models. In a second step, they were exposed to human oral environment *in situ* in order to explore potential application in dentistry. *In vitro*, the cuticular structures were found to resist wetting by albumin solutions for up to 3 h and colonization by *Staphylococcus epidermidis* was inhibited. When exposed in the oral cavity, initial pellicle formation was of high heterogeneity: parts of the surface were coated by adsorbed proteins, others remained uncoated but exhibited locally attached, ‘bridging’, proteinaceous membranes spanning across cavities of the cuticle surface; this unique phenomenon was observed for the first time. Also the degree of bacterial colonization varied considerably. In conclusion, the springtail cuticle partially modulates bioadhesion in the oral cavity in a unique and specific manner, but it has no universal effect. Especially after longer exposure, the nanotextured surface of springtails is masked by the pellicle, resulting in subsequent bacterial colonization, and, thus, cannot effectively avoid bioadhesion in the oral cavity comprehensively. Nevertheless, the observed phenomena offer valuable information and new perspectives for the development of antifouling surfaces applicable in the oral cavity.

## 1. Introduction

**Figure 1.**
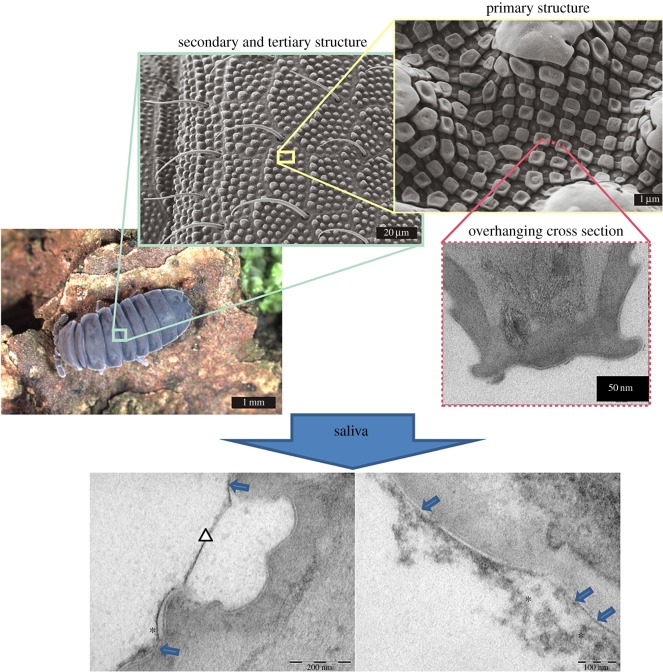
Cuticular structure of the springtail *Tetrodontophora bielanensis* (Collembola, Hexapoda). Note the characteristic nanostructure of these hexapods' dorsum with mushroom like pins and small cavities arranged in a comb-like pattern. Preliminary *in vitro* experiments with human saliva indicated interesting so far unknown phenomena of bioadhesion (transmission electron microscopy analysis). Pellicle formation (*) under *in vitro* conditions after 30-min exposure in centrifuged and sterile filtered human saliva. In direct contact to the surface, the pellicle is arranged as an electron dense less than 10 nm thick basal layer (blue arrows), partially covered by an up to 200 nm thick loosely arranged pellicle layer. Interestingly, cavities between pits on the springtail's surface are covered by membrane-like structures (white triangle) due to the *in vitro* pellicle formation. These observations gave the impulse for further experiments.

The nanotextured cuticle of springtails (Collembola) [1] has been described as a mechanically stable, highly repellant and self-cleaning surface, which prevents biofilm formation even under the challenging conditions of the soil [1]. Springtails are the most widespread group of hexapods [2,3]. Respiring through their cuticle, they have evolved a non-adhesive surface that exhibits a unique hierarchical structure consisting of microscaled bristles and granules (tertiary and secondary structure) and a comb-like alignment of smaller mushroom-shaped granules with interconnecting ridges (primary structure) (figure 1). As reported previously, the liquid repellency of springtails is perpetuated for low-surface tension liquids and under high pressure [1]. The strong wetting resistance was shown to be caused by the mushroom-like overhanging cross sections of the cuticular nanostructure irrespective of the solid materials [4,5]. Incorporation of these cross sections into rhombic or hexagonal alignments of comb-like cavities leads to mechanical stability, which makes it interesting for antifouling applications.

Caries and periodontitis are still the most relevant oral diseases and represent considerable challenges in dental research. Both are induced by adherent biofilms on non-shedding surfaces, the dental hard tissues as well as dental restorative and implant materials [6,7]. The first step of bioadhesion in the challenging and specific oral environment is the formation of the so-called pellicle layer which is composed of proteins, glycoproteins and lipids [7]. Its ultrastructure is characterized by a thin electron dense basal layer (10–20 nm in thickness) which is formed almost instantaneously on all solid surfaces exposed to the oral fluids [7]. The adsorption of the biomolecules is a complex process driven by different physicochemical interactions yielding a gain in entropy. In a second step, globular and granular structures adsorb to this basal layer [8]. The final thickness reached by the pellicle within 2–6 h varies between 30 and 750 nm depending on the location in the oral cavity [8]. The pellicle on all common dental materials is of high uniformity and seems to equalize differences of the intrinsic physicochemical surface properties of the applied materials [7]. Though the pellicle serves as a lubricant and protective layer on dental surfaces [8], bacterial colonization of the teeth and dental material starts within hours [8]. Accordingly, there is a persisting need for non-adhesive surfaces that are effective and applicable in the oral cavity for coatings of teeth and dental materials [9].

Various types of nano- and microtextured surfaces have been considered for biofilm management in dentistry [10–12], however, to the best knowledge of the authors, without translation into widely applied products. This might be attributed to the inevitable pellicle formation masking nanostructures but also to the destruction of the structures by mechanical forces associated with the application in the oral cavity. Non-adhesive biological design strategies might be suitable as a starting point for the development of surface coatings to be applied in preventive dentistry. Owing to the rather simple accessibility as well as to the challenging conditions, the oral cavity is an excellent *in vivo* model to evaluate new strategies for potential application in humans [7]. The nanotextured cuticle of springtails or biomimetic structures mimicking this unique nanostructure might be suitable for application in dentistry and in medicine.

Accordingly, the aim of the present study was to further characterize surface interactions and bioadhesion phenomena on springtails, shed cuticles (exuviae) and cuticle replicates made of poly(ethylene glycol) diacrylate (PEGda) *in vitro* adopting well accepted experimental set-ups based on typical and clinically relevant model bacteria [13–15] in order to understand the unique phenomena occurring on Collembola. The *in vitro* experiments served as a reference. Furthermore, *in vitro* pilot experiments indicated unique interactions of collembolan with human saliva (figure 1). Owing to this fact, in a second step springtails and replicates were exposed to the oral fluids *in situ*. It was hypothesized that bioadhesion on springtails and biomimetic replicates in the oral cavity differs from pellicle formation and bacterial adhesion on dental hard tissues yielding reduced biofilm formation for potential application in dentistry.

## 2. Methods

### 2.1. Specimens and replicates

The springtails (*Tetrodontophora bielanensis*) were collected in the wooded mountains of Saxony close to Dresden, Germany. They were kept in a small terrarium until needed for the experiments. Directly before bacterial and oral experiments, springtails were sacrificed by deep-freezing for 15 min. For bacterial *in vitro* assays the animals were fixed on glass slides by partially dipping them ventrally in a prepolymer solution (Fluorolink MD 700, Solvay, Hannover, Germany) and subsequent UV-curing of the polymer on the glass. Half of the fixed samples were immersed in water and exposed to high pressure (approx. 6 bar) to force a full wetting of the cuticle prior to bacterial exposure. For protein adsorption tests, shed cuticles (exuviae) were collected in the terrarium and mounted on cover slides.

Replicates were manufactured by using perfluoropolyether dimethacrylate Fluorolink MD 40 (PFPE, Solvay, Hannover, Germany) for the first moulding of the negative stamp and PEGda (Mn = 700, Sigma Aldrich, Munich, Germany) for the second moulding of the final replication. Both pre-polymers contained 0.5 wt% Irgacure 651 (Ciba, Basel, Switzerland) and were cross-linked by UV irradiation under nitrogen atmosphere. The detailed procedure has been described previously [2]. The replication of the PFPE stamp with the PEGda precursor solution includes degassing before the UV-curing of the PEGda. To obtain replicates with all structural details and some without the nanostructured features, the samples were degassed for 12 h and 10 min, respectively. One half of the animal was dipped in fluid monomer polymerized afterwards. The uncoated and relevant dorsum of the animal was replicated in the following. Accordingly, potential modification of the cuticle could be excluded [4].

### 2.2. First step: common *in vitro* models

#### 2.2.1. Protein adsorption

Exuviae from *T. bielanensis* were taken from the litter substrate in the terrarium and deposited in MilliQ, where they float and spread on the liquid–gas boundary with the hydrophobic, structured side facing upwards. The floating cuticles were then drawn on cover slides and enclosed by glass cylinders with inner diameters of 7 mm and heights of 9 mm. The glass cylinders were mounted with silicone adhesive (NUSIL, Carpinteria, CA, USA) on these cover slides encasing the attached cuticles. The volume was filled with a solution of TRITC-labelled bovine serum albumin (BSA; Sigma Aldrich, Munich, Germany) dissolved in the phosphate buffer and protein concentrations of 0.1 and 2 mg ml^−1^ [16]. The volume was sealed with a second cover and oil on top of the glass cylinder. The protein adsorption on the cuticle was tracked by time lapse using the fluorescence signal of the protein, and the reflection signal at the liquid–gas interfaces of the trapped air at the cuticle [17].

#### 2.2.2. Bacterial assays

*Staphylococcus epidermidis* (strain ATCC 12228) were grown overnight in Luria-Bertani medium [15]. The culture was washed three times by centrifuging, removing the supernatant and resuspension in a fresh medium. The cell density (OD_600_) was adjusted to 0.0075 for 24 h and to 0.75 for 2 min and 30 min. After incubation under gentle shaking, the springtails and the replicates were fixed in paraformaldehyde, washed with phosphate-buffered saline and MilliQ, and dried with nitrogen. The experiments were performed three times with at least two samples per condition.

#### 2.2.3. Scanning electron microscopy

For scanning electron microscopy (SEM) imaging, the samples (springtails, replicates and exuviae) were dried and sputter-coated with 10 nm gold (BALZERS SCD 050 Sputter Coater). The animals and their exuviae could be dried under normal conditions, whereas the replicates had to be dried in an exsiccator under vacuum pumping for 16 h due to the strong uptake of water in the PEGda. Bacteria were counted in at least nine areas per sample type by the detailed analysis of all connected bacteria referred to as colonies. From these data the colony density and the size distribution of colonies were evaluated in order to illustrate more than the amount of cells with the intrinsic strong deviations due to partially inhomogeneous cell coverage, i.e. large areas without and large areas with many cells.

### 2.3. Second step: oral exposure

*In situ* experiments were conducted as described previously [18–20]. Springtails were fixed to individual upper jaw splints with silicone impression material and exposed to the oral fluids for 3 min, 120 min, 8 h and 24 h, respectively, by three volunteers (members of the laboratory staff, authors of the paper). The dorsum of the insects was the object of the study. Bovine enamel slabs served as a reference. They were prepared and polished as described previously [19–21].

#### 2.3.1. Transmission electron microscopy

After oral exposure, springtail samples as well as enamel specimens were fixed in 2.5% glutaraldehyde/1.5% formaldehyde for 2 h. Specimens were rinsed in cacodylate buffer, and post-fixation was performed by 2 h exposure in 1% osmium tetroxide. Subsequently, all samples were dehydrated by an ascending series of alcohol and embedded in Araldite CY 212 (Plano, Wetzlar, Germany). The embedded enamel specimens were demineralized in 1 M hydrochloric acid in order to completely remove the enamel and re-embedded in Araldite CY 212. Ultrathin sections of pellicles and biofilms formed *in situ* on enamel as well as on springtails were cut with a diamond knife (Mikrostar 458, Mikrotechnik, Bensheim, Germany), mounted in an Ultra-Cut-E microtome (Reichert, Heidelberg, Germany). The ultrathin sections were contrasted with uranyl acetate and lead citrate, and analysed in a TECNAI 12 Biotwin transmission electron microscope (FEI, Eindhoven, The Netherlands). Characteristic and representative images of the pellicle layers and biofilms were taken at magnifications of up to 100 000-fold.

#### 2.3.2. 4′,6-Diamidino-2-phenylindole staining

4′,6-Diamidino-2-phenylindole (DAPI) staining was performed directly after oral exposure as described previously [21,22]. The fluorescence–microscopic evaluation was carried out in different levels, the digital images were overlaid.

## 3. Results

### 3.1. Common *in vitro* models

#### 3.1.1. Protein adsorption on shed cuticles

Protein adsorption experiments on exuviae were performed to evaluate the resistance against wetting under more demanding conditions compared to previous applications of ordinary low-surface tension liquids. The presence of proteins is known to induce short- and long-term changes of the interfacial energies in the three-phase system (solid–liquid–gas) due to an immediate adsorption and permanent exchange of molecules on all liquid–gas and liquid–solid interfaces [23,24]. Therefore, static wetting states cannot be expected because of high molecular dynamics of the proteins at the three-phase line, i.e. a dynamic wetting front.

**Figure 2.**
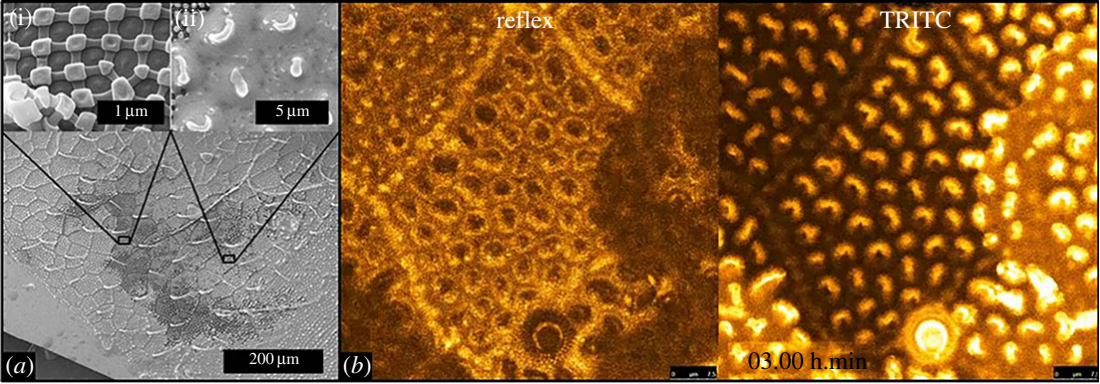
Wetting of springtail cuticle by the BSA solution. (*a*) SEM image of an exuviae exposed to the BSA solution for 4 h. The upper insets show non-clogged (i) and clogged areas (ii). (*b*) Fluorescence imaging: wetting of collembolan cuticle after 3 h with 2 mg ml^−1^ BSA in phosphate-buffered saline. The bright areas in the left image (‘reflex’ means reflection) represent the reflection signal of the liquid–gas boundary above a still non-wetted part of the cuticle, whereas the bright areas in the right image represent the fluorescence signal of adsorbed BSA on the wetted cuticle (electronic supplementary material, movie S1).

The SEM imaging of the ultrathin exuviae (thickness approx. 100 nm) revealed a collapsed secondary and tertiary structure (figure 1), due to the missing mechanical support of an underlying exoskeleton, but the primary nanostructure exhibits all structural details found on unshed springtail cuticles. The wetting experiments with BSA containing a phosphate buffer solution were performed with low and high protein concentrations (0.1 and 2 mg ml^−1^). Irrespective of the concentration, fluorescence imaging has shown that BSA solution quickly wets the majority of the exuviae during the first minute (figure 2), but a few remaining, more resistant, parts could inhibit a full wetting and associated protein adsorption for more than 3 h (figure 2*b*; electronic supplementary material, movie SM1). The wetting resistance and the advancing wetting of the exuviae by the protein solution were deduced from the change of the reflection signal from the liquid–gas boundary and the actual fluorescence signal of the labelled BSA. Both signals have shown mostly opponent relative intensities and dynamics. Areas with increasing fluorescence intensities, related to wetting and protein adsorption, simultaneously exhibit decreasing reflection signals due to a vanishing liquid–gas interface. Areas of the exuviae which are wetted by the protein solution have shown a strong clogging of the primary structure in SEM (figure 2*a*). However, in some cases unclogged cuticular areas could be observed even after two weeks of exposure to the protein solution, although after this time scale fluorescence imaging has no longer given any indication for a maintained wetting resistance, i.e. very low reflection signal and high fluorescence signal from all parts of the structure.

#### 3.1.2. Bacterial assays with springtails and replicas *in vitro*

**Figure 3.**
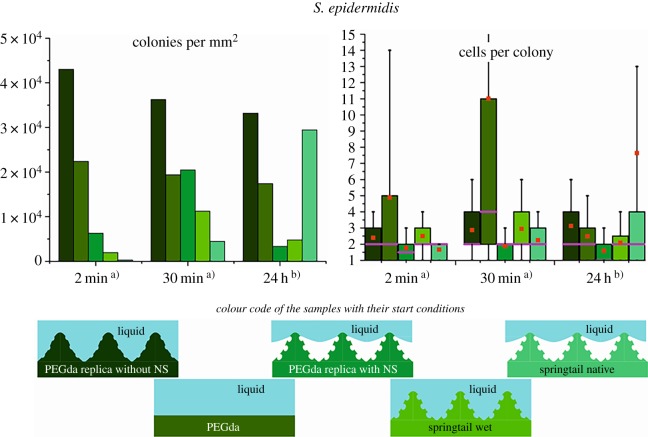
Bacterial assays on springtails and their polymeric replicates. Note that the start conditions of the cell density are 100× smaller for the 24 h (6 × 10^6^ cells ml^−1^) assays than for the short-term assays of 2 min and 30 min (6 × 10^8^ cells ml^−1^). The distribution of cells per colony is presented in box–whisker plots with half of the data points within the box and 80% within the whiskers. Pink lines and red square dots mark the median and the mean values, respectively. Representative SEM images are given in electronic supplementary material, fig. S1. NS, nanostructural features.

For the bacterial assays, half of the analysed animals were forced to a full wetting of the primary nanostructure by the application of high pressure within a pressure chamber filled with water (greater than 5 bar) in order to elucidate the actual anti-bioadhesion effect of the wetting resistance compared to the pure topographical interaction of the cuticle in direct contact with bacterial cells. Furthermore, replications of the cuticular surface allowed clarification of structural impacts on bacterial colonization by fabrication of polymeric copies with detailed primary nanostructure and without the primary nanostructure and unstructured references of the same material (PEGda). The amount of adherent bacteria on the tested substrates (figure 3) is represented by the colony densities and the detailed respective size distributions of all counted colonies on each sample type. This provides information on the amount of cells, the average cell clustering and the homogeneity of the clustering. The amount of adherent cells can be considered as the colony density (‘colonies per mm²’ in figure 3, left) multiplied with the mean value of the colony size distribution (red square dot in the whisker–box diagram ‘cells per colony’ in figure 3, right), but an illustration of the amount of cells in one plot would show misleading strong deviations due to partially very inhomogeneous surface coverage between different areas on the samples.

In the initial phase of adhesion, non-wetted springtails clearly inhibit bacterial colonization, i.e. almost no cells could be found after 2 min and clearly fewer than on all other samples after 30 min (figure 3; electronic supplementary material, figure S1). Wetted cuticles, forced by high pressure, show clearly more attached cells after 30 min than the non-wetted one, but significantly fewer than unstructured PEGda (larger colony size and higher density of colonies) and replicates without the nanostructural details (similar colony size but much higher density of colonies). On replicates with all nanostructural features a low amount of adherent bacteria, similar to the wetted cuticles (smaller colony density but higher colony size), could be found.

After 24 h on the previously non-wetted springtail cuticle with almost no cells at the beginning, the strongest colonization was observed. The cells were highly clustered, but the clustering was very inhomogeneous. There were areas of at least 1000 µm^2^ free of cells or with few single and double cells, surrounded by clusters of big colonies. The clearly lowest amount of cells after 24 h was found on PEGda replicas with all structural details. However, surprisingly even the fully wetted springtails have shown a low amount of adherent cells, i.e. much lower than previously non-wetted cuticles.

Pilot experiments with springtails incubated in human saliva gave the impulse for *in situ* experiments in order to evaluate the efficacy of the nanotopography for modulation of bioadhesion in the oral cavity (figure 1).

### 3.2. *In situ* experiments in the oral cavity

**Figure 4.**
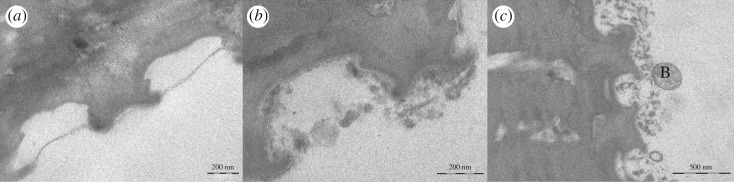
Pellicle formation on springtails *in situ* (in the oral cavity). After 3 min (*a*,*b*), sometimes the formation of membrane-like structures was observed, covering the cavities between the pits. Partial association of granular and globular protein aggregates with the springtails' surface occurred on top of the pits and within the cavities between pits. In general, the typical continuous electron dense basal pellicle which is typical for the dental pellicle was not detectable unequivocally. After 120 min (*c*), more pronounced adsorption of granular and globular structures on the pit's surface and within the cavities was observed. However, this was not a homogeneous process. Note adherence of first bacteria (B).

**Figure 5.**
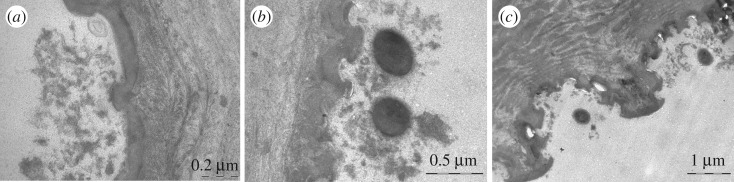
Initial bacterial colonization of springtails in the oral cavity. After 8 h of oral exposure (*a*–*c*), more bacteria were detectable. Note the inhomogeneity of pellicle formation by adsorption of globularly and granularly shaped protein aggregates forming network-like structures. Bacteria are embedded in the pellicle layer or adhere to the pellicle surface. Sometimes, bacteria seemed to prefer the large-scale cavities of the springtail surface.

**Figure 6.**
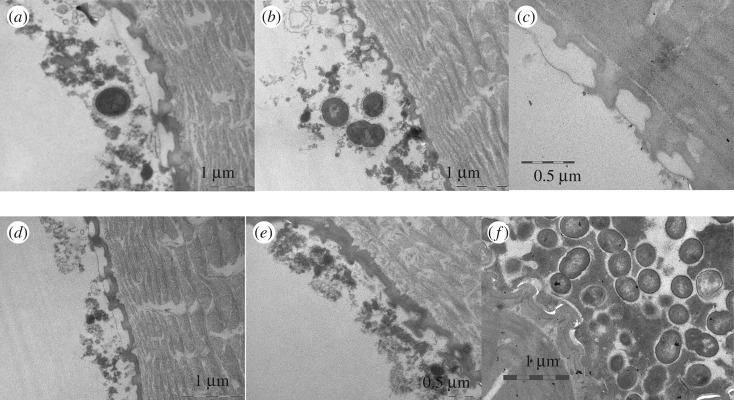
Progressing biofilm formation on springtails exposed to the oral fluids for 24 h. The pattern of bacterial colonization varied considerably (*a*–*f*). On some samples, membrane-like bridging of the cavities by adsorbed protein layers was observed (*a*,*c*,*d*), combined with very low level of bacterial colonization. Others yielded filling of the cavities by adsorbed protein aggregates (*e*) and extensive bacterial biofilm formation (*f*). Irrespective of the bacterial colonization also the ultrastructure of the underlying pellicle varied considerably. Globular and granular structures of differing electron density were observed, and the pellicle was of very different tenacity as indicated by the varying pattern of attachment to the springtails' surface.

The *in situ* trials indicated multifaceted patterns of initial and progressive bioadhesion on springtails (figures 4–6). Bioadhesion on dental enamel served as a reference (electronic supplementary material, figure S2). Pellicle formation and initial bacterial colonization of enamel slabs took place in a well-known process [25]. Every surface was coated almost instantaneously by an electron dense basal pellicle of high tenacity (5–10 nm in thickness). In the following, granular and globular structures adsorbed to this layer. First bacteria were detected within 120 min, but pronounced bacterial colonization mainly started after 8 h. Initial biofilm formation was observed after 24 h.

On springtails exposed to the oral fluids, a wide and inhomogeneous spectrum of phenomena was observed concerning the different steps of bioadhesion and biofilm formation. In part, it corresponded to the observations made on dental enamel; sometimes, protein adsorption and bacterial colonization were even more pronounced. Thereby the topography of the surface seemed to facilitate bioadsorption and the accumulation of proteinaceous globular structures as well as of bacteria and extracellular matrix. By contrast, some sections of the samples yielded a completely different and up to now unknown pattern of bioadhesion. This applied especially for the initial phase. Some cavities of the springtails' surfaces were covered by membrane-like pellicle structures. Granular and globular structures were completely missing or of very low density when compared with the enamel samples. In part, the pellicle formed membrane-like structures. Some parts of the springtails were completely free of bacteria.

Interestingly, these unique phenomena were not observed on replicates. In contrast to the animals, pronounced pellicle formation and bacterial colonization were observed on the textured replicates *in situ*.

To get an overview on the mode of bacterial colonization, fluorescence microscopic imaging was conducted additionally (electronic supplementary material, figures S3 and S4). DAPI staining does not require extensive washes and fixation procedures. Accordingly loosely attached bacteria which might be lost during the preparation for transmission electron microscopy or SEM are visualized. A considerable number of bacteria were visible on the nanostructured dorsum of the springtails. However, on the surfaces of real animals fewer bacteria were accumulated than on polished enamel surfaces, whereas replicates were colonized extensively by bacteria within 8 h.

## 4. Discussion

The springtail cuticle represents an impressive example of non-adhesive surface nanomorphology, effectively protecting the organism in its natural environment, the soil [1]. In this study, springtails, shed cuticles (exuviae) and cuticle replicates made of PEGda were studied after exposure to protein solutions and bacterial cultures *in vitro* as well as to the human oral environment *in situ*. To the best knowledge of the authors, every solid surface in the oral cavity is coated by a continuous proteinaceous pellicle layer [7]. This applies also for polytetrafluoroethylene and specifically designed theta surfaces [7,26]. However, on springtail cuticle surfaces after exposure to the human oral cavity, membrane-like structures covering the small cavities of the springtail nanostructure were observed which were not found after similar treatment of synthetic replicates of the cuticle. This suggests a combination of nanotopography and surface chemistry to cooperatively yield the particular characteristics of the cuticle. *In vitro* experiments were performed to understand the mechanisms of bioadhesion on cuticles and to verify this hypothesis. Incubation with *Staphylococcus epidermidis* seemed to confirm the postulate because springtails lost their anti-adhesive properties after 24 h, likely due to degradation. Interestingly, this effect was not observed with the wetted cuticles *in vitro*.

In either case, the ubiquitous pellicle formation in the oral cavity [7,8] known to equalize the physicochemical properties of very different surfaces, also ultimately resulted in the bacterial colonization of large areas of the springtail cuticle. This underlines that the oral cavity is a specific and extremely challenging environment [7]. Strategies working very well in other systems lose their antifouling properties due to the highly effective adsorption of proteins and glycoproteins from the saliva. This is confirmed by the results of our *in vitro* experiments where much better antifouling effects have been observed. In particular, protein adsorption patterns from oral fluids clearly differed from the results of the widely used BSA adsorption assay [27].

The oral fluids are much more complex than pure albumin solutions. Even the saliva itself contains more than 1000 different proteins and peptides [27]. *In situ* experiments are therefore critically important to evaluate materials intended for application in the oral cavity or generally in humans.

Irrespective of the observations in the oral cavity, the collected data provide new insights into the operational principle constituting the non-adhesive characteristics of springtail cuticles. It resists bacterial adhesion in many conditions. This might be attributed to the sub-cell sized dimensions of the primary structures. Artificial periodic surface structures with features in the range of those cuticular primary structures ( approx. 500 nm) were similarly shown to inhibit bacterial adhesion [15]. In addition, the formation of free standing protein layers observed after oral cavity exposure, spanning across the nanocavities of the cuticle and including the adsorption of biomolecules at the air–water interface, may contribute to the peculiarities of the springtail cuticle.

It has been supposed that ‘the development of biofilm resistant materials will likely require integrated approaches combining chemical, mechanical, and topographical elements into the design of surfaces and interfaces' [28]. The present study clearly underlines this statement.

## 5. Conclusion

The springtail cuticle, a highly effective omniphobic surface, was confirmed to exhibit non-adhesive characteristics when tested in standard serum albumin and bacterial adhesion assays *in vitro*. When exposed to the complex oral environment *in situ*, to some extent, unique phenomena of protein adsorption and bioadhesion have been observed for the first time indicating modulated surface interactions. However, especially after longer exposure, the nanotextured surface of springtails is masked by the pellicle, resulting in subsequent bacterial colonization, and, thus, cannot effectively avoid bioadhesion in the oral cavity comprehensively in a sustainable manner. Nevertheless, the observations offer valuable information and new perspectives for the development of antifouling surfaces applicable in humans. In this context, further studies are required based on *in situ* experiments considering the specific conditions in the oral cavity.

## Supplementary Material

Supplement figures 1 - 4

## Supplementary Material

Data for figure 3

## Data accessibility

Most of the study is based on different imaging techniques. Relevant images are included in the paper or in the electronic supplementary material. Raw data/basic data of the *in vitro* experiments (figure 3) are included in electronic supplementary material, table S1.
