# Supplementary material for: Impact of the springtail's cuticle nanotopography on bioadhesion and biofilm formation in vitro and in the oral cavity
Source: R Soc Open Sci. 2018 Jul 4;5(7):171742. doi: 10.1098/rsos.171742 (PMC6083677; doi:10.1098/rsos.171742)
Supplement: Supplement figures 1 - 4 [file rsos171742supp1.docx]

**Supporting Information/ supplement**

Impact of the springtail´s cuticle nanotopography on bioadhesion and biofilm formation in vitro and in the oral cavity

Christian Hannig^1*^, Ralf Helbig^2^, Julia Hilsenbeck^2^, Carsten Werner^2^, Matthias Hannig^3^

*corresponding author

[Christian.hannig@uniklinikum-dresden.de](mailto:Christian.hannig@uniklinikum-dresden.de), Clinic of Operative and Pediatric Dentistry, Medical Faculty Carl Gustav Carus, Technische Universität Dresden, Fetscherstraße 74, D-01307 Dresden, Germany

**Tel. +49 (0)351 458-2713**

**Fax +49 (0)351 458-5381**

**^1^ Clinic of Operative and Pediatric Dentistry, Medical Faculty Carl Gustav Carus, Technische Universität Dresden, Fetscherstraße 74, D-01307 Dresden, Germany**

**^2^ Leibniz-Institut für Polymerforschung Dresden e.V., Max Bergmann Center of Biomaterials, Hohe Strasse 6, D-01069 Dresden, Germany**

**^3^ Clinic of Operative Dentistry, Periodontology and Preventive Dentistry, University Hospital, Saarland University, Building 73, D- 66421 Homburg/Saar, Germany**

**Movie – see separate file**

**
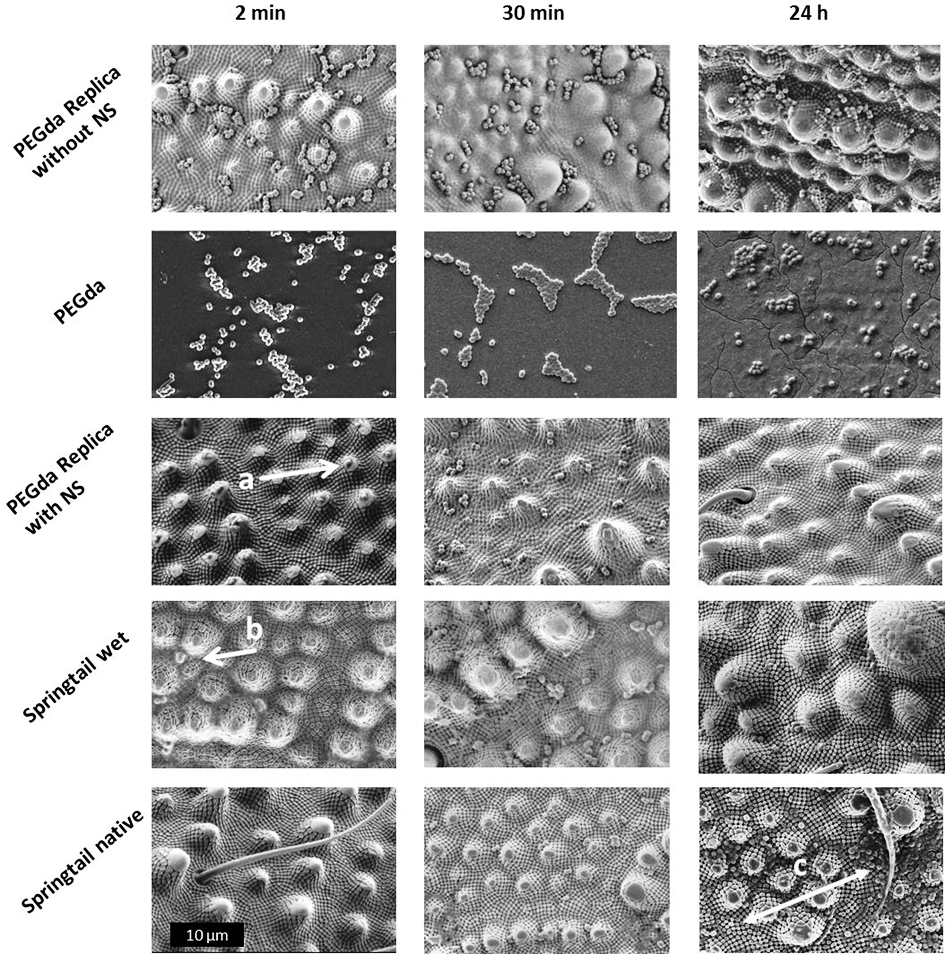
Fig. S1:** Representative SEM images of *S. epidermidis* on springtail cuticles and their polymeric
replicates. The replicas are made of PEGda with and without the nanostructural (NS) features. Springtails were exposed to bacterial media in completely wetted and unwetted
state (wet and native respectively) at the start of the experiments. a) bacterial cells on top of the secondary structure is an evidence for a pronounced plastron. b ) cells mainly onto the primary structures as a result of a full wetted surface. c) strong variations of surface coverage (no bacteria vs large colonies).


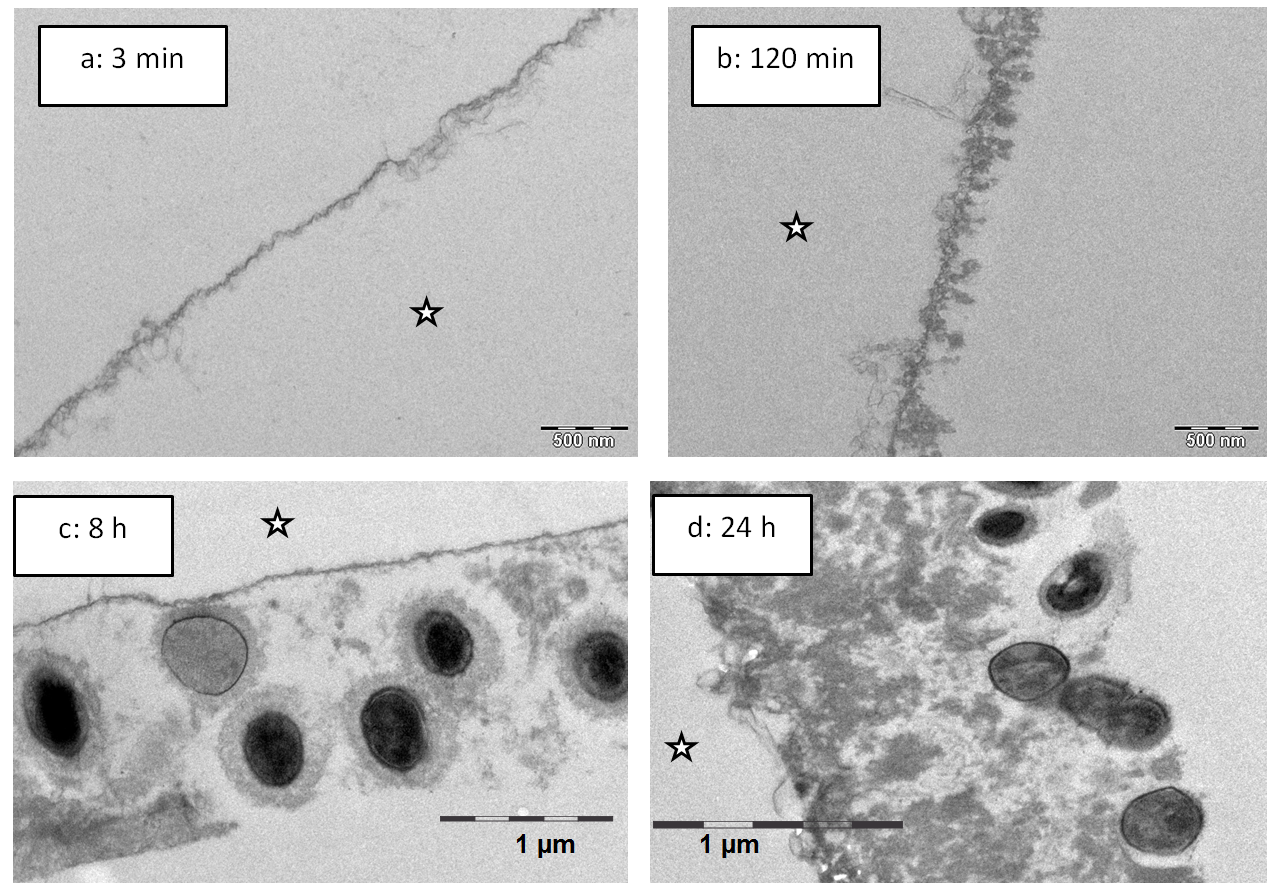


**Fig. S2: Bioadhesion on control specimens *in situ* (dental enamel).** After 3 min, there is a thin electron dense pellicle layer. After 120 min, the ubiquitous electron dense basal pellicle is covered by granular and globular structures. Initial bacterial colonization can be observed after 8 h. The enamel itself was removed during preparation of the samples. The former enamel site is marked with an asterisk.

**
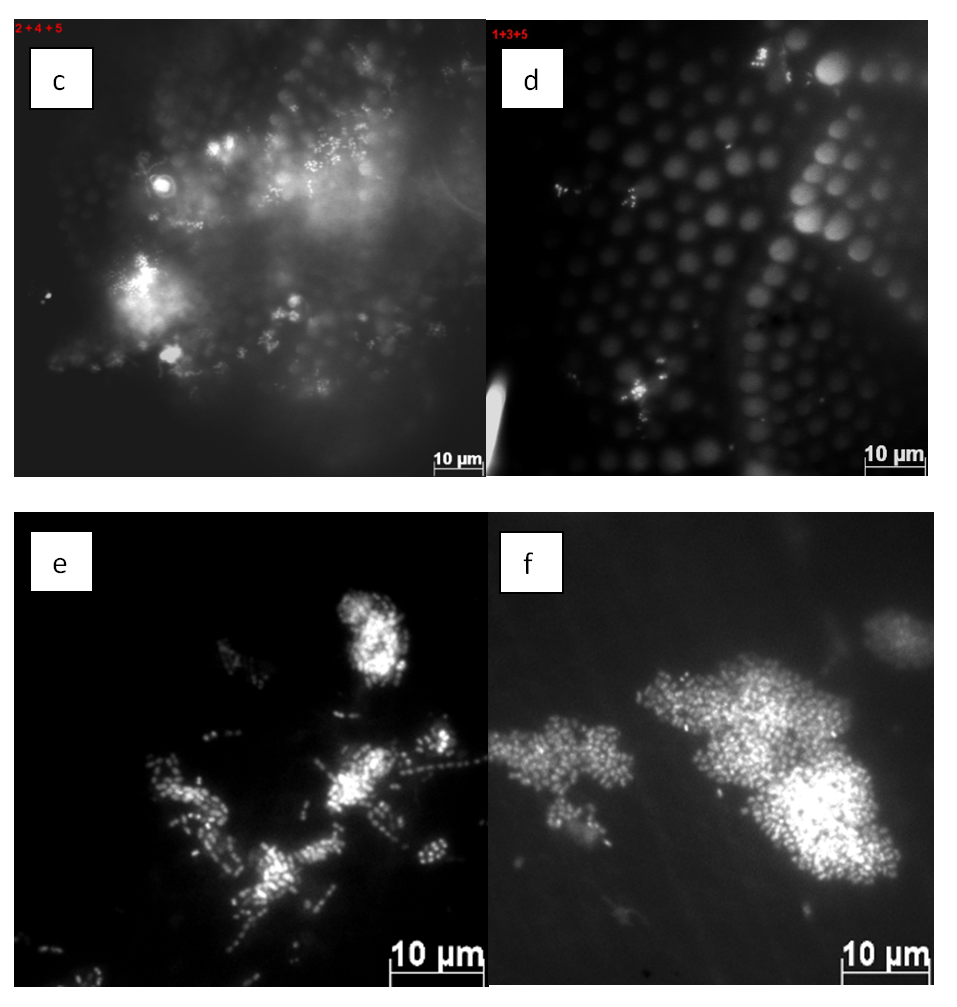
Fig. S3: DAPI-staining, native springtails after 8 h of oral exposure**: the fluorescence microscopic evaluation yielded of adherent bacteria; a, b: single cutting planes; b, c: combination of cutting planes. The typical nanostructure of the springtails is visualized as white dots. Please note the different optical properties of replicates (fig. 10) and springtails. Polished enamel slabs worn by the same subjects for 8 h showed higher numbers of adherent bacteria (e, f).

**
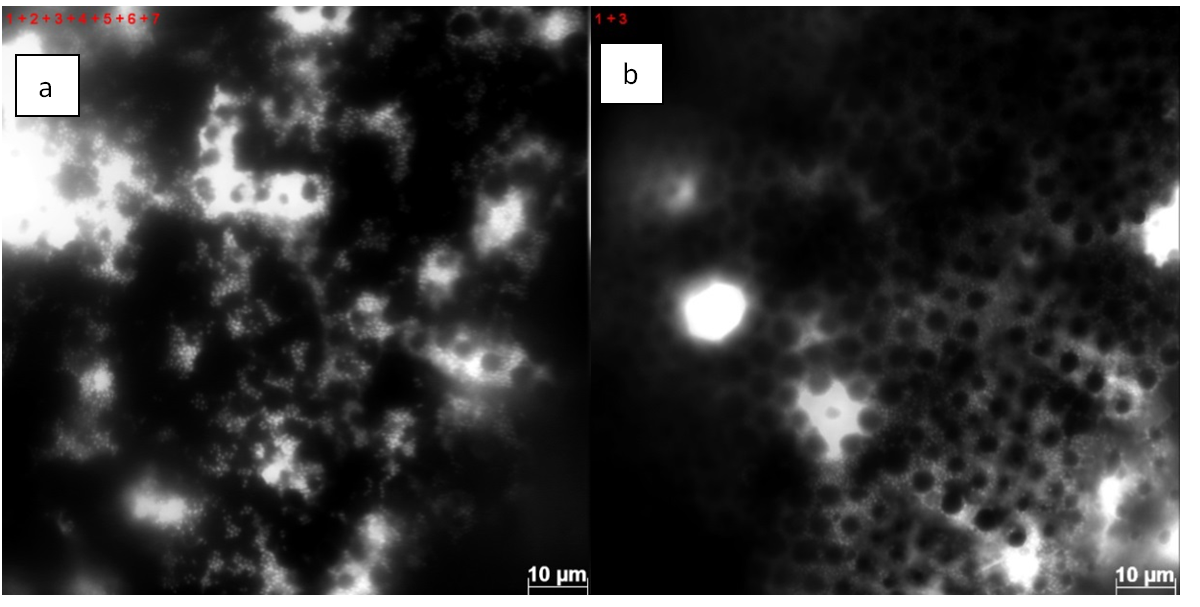
Fig. S4: DAPI-staining, replicates:** there was considerably higher accumulation of bacteria on the replicates than on the native springtails; a: cumulative depiction of 7 layers, 8 h of oral exposure; b: combination of two layers. The typical nanostructure of the springtails is visualized as black dots. Please note the different optical properties of replicates and springtails.
